# Hierarchical multistate models from population data: an application to parity statuses

**DOI:** 10.7717/peerj.2535

**Published:** 2016-09-29

**Authors:** Robert Schoen

**Affiliations:** Population Research Institute, Pennsylvania State University, University Park, PA, United States of America

**Keywords:** Multistate models, Parity status life tables, Childlessness, Hierarchical models, Sequential cross-sections, Polytrees

## Abstract

Hierarchical models are characterized by having *N* living states connected by *N* − 1 rates of transfer. Demographic measures for such models can be calculated directly from counts of the number of persons in each state at two nearby points in time. Exploiting the ability of population stocks to determine the flows in hierarchical models expands the range of demographic analysis. The value of such analyses is illustrated by an application to childbearing, where the states of interest reflect the number of children a woman has born. Using Census data on the distribution of women by age and parity, a parity status life table for US Women, 2005–2010, is constructed. That analysis shows that nearly a quarter of American women are likely to remain childless, with a 0–3 child pattern replacing the 2–4 child pattern of the past.

## Introduction

Multistate population models are widely used in demographic analyses, as they can reflect any number of states and any pattern of movements between those states. The first multistate analysis is apparently due to DuPasquier (1912), but multistate analyses received little attention until the 1970s (e.g., Rogers, 1975). In the 1980s, their nature and significance to demography was well established (Land & Rogers, 1982; Schoen, 1988). In general, a model with *N* living states has *N*^2^ possible transfers: *N*(*N* − 1) between living states, and *N* from each state to the “dead” state.

Population data on the number of persons in each state at the beginning and end of an age/time interval can provide only *N* equations, *N* − 1 for transfers between living states and 1 for the overall rate of death. Various procedures have been advanced to estimate all of the elements of the matrix that transforms the population at the beginning of an interval into the population at the end of the interval.

The leading technique is iterative proportional fitting (IPF), also known as the Deming-Stephan procedure, bi-proportional filling, and the RAS method (Bishop, Fienberg & Holland, 1975; Willekens, 1982). IPF yields the solution that maximizes entropy, i.e., it finds the transfers that can occur in the greatest number of ways. Schoen & Jonsson (2003) advanced an alternative, more demographic, approach based on estimating changes in the attractiveness of model states. More recently, Schoen (2016) presented a Quadratic Estimation of Rates of Transfer (QERT) procedure, based on the assumption that the products of selected pairs of rates can be assumed constant. All three approaches assume a pattern that is believed to characterize the interstate movements, and thus produce estimated rather than calculated values.

This article focuses on “hierarchical” multistate models, that is on *N* state models with only *N* − 1 possible transfers connecting the living states and 1 unknown risk of death. In hierarchical models, a direct algebraic solution for the transfer matrix is possible, without the need to assume any pattern of interstate movements. We first discuss the parallels between hierarchical models and graphs, then present techniques for using sequential population data to calculate rates of transfer and transition probabilities, and proceed to apply the approach to calculate a Parity Status Life Table for US Women, 2005–10.

## The nature of the hierarchical model

The *N* living states of a hierarchical model are linked by *N* − 1 occurrence/exposure rates of the form *m*_*ij*_, where *i* is the origin state and *j* is the destination state. The dead state is indicated by the symbol *δ*. For every living state there is at least one connection to another living state, though all states are not necessarily reachable from any given state. For example, consider a model with two living states, A and B. With interstate transfer rate *m*_AB_, it is possible to go from state A to state B, but not from state B to state A. The hierarchical model greatly restricts the range of possible interstate movements. It is a special case of the general multistate model, but one where the rates over an interval can be found from the populations at the beginning and end of the interval.

Graph theory can provide some useful nomenclature and context. The living states of a multistate model correspond to the vertices or nodes of a graph, and the rates connecting them are the edges or connecting lines of the graph. Hierarchical multistate models are connected graphs with *N* vertices and *N* − 1 edges, and hence constitute a “tree” (Chartrand & Zhang, 2012). Since the rates take persons from one specified state to another, the tree is “oriented” or, using the term coined by Rebane & Pearl (1987), a “*polytree*.”

Graph theorists have explored the number of distinct polytrees, or distinct multistate configurations, in models with *N* states (or vertices). When *N* = 2, there is only one form, with the single transfer rate *m*_12_. When *N* = 3, there are 3 forms, which can be described by the rate pairs (*m*_12_, *m*_23_), (*m*_12_, *m*_13_), and (*m*_13_, *m*_23_). There is no general rule for the number of forms, but when *N* = 4 there are 8 forms, when *N* = 5 there are 27 forms, and when *N* = 6 there are 91 forms (Chartrand & Zhang, 2012). Polytrees thus encompass a substantial number of distinct hierarchical multistate models.

Polytrees have demographic significance because transfers in every multistate model that is a polytree can be fully determined from knowledge of the population, by state, at the beginning and end of every interval of observation. Such multistate models can be used to analyze of a wide range of demographic behaviors, including parity progression, educational attainment, career trajectories, and the spread of infections. A procedure for carrying out the calculations needed to produce a hierarchical multistate life table from cross-sectional data is presented in the next sections.

## Finding multistate rates from beginning and ending populations

With *N* states, the beginning and ending populations support *N* equations that describe the flows (movements) between model states. Those *N* equations can be used to determine the *N* − 1 rates of transfer between those living states, and an overall rate of transfer to death. Each distinct hierarchical model (polytree) has a different set of *N* flow equations, but all those sets of equations are readily solvable because their *N* equations are independent.

For example, consider the case where *N* = 3 and the polytree form is (*m*_12_, *m*_13_). The flow equations can then be written

\begin{eqnarray*}{P}_{1}(x+n,n,t+n)={P}_{1}(x,n,t)-P{P}_{1}(x,n,t)[{m}_{\delta }(x,n,t)+{m}_{12}(x,n,t)+{m}_{13}(x,n,t)] \end{eqnarray*}

\begin{eqnarray*}{P}_{2}(x+n,n,t+n)={P}_{2}(x,n,t)-P{P}_{2}(x,n,t){m}_{\delta }(x,n,t)+P{P}_{1}(x,n,t){m}_{12}(x,n,t) \end{eqnarray*}

(1) \begin{eqnarray*}{P}_{3}(x+n,n,t+n)={P}_{3}(x,n,t)-P{P}_{3}(x,n,t){m}_{\delta }(x,n,t)+P{P}_{1}(x,n,t){m}_{13}(x,n,t)\end{eqnarray*}

where *P*_*j*_(*x*, *n*, *t*) denotes the number of persons in state *j* between the ages of *x* and *x* + *n* at time *t*, and *PP*_*j*_(*x*, *n*, *t*) is the number of person-years lived in state *j* between times *t* and *t* + *n* by persons aged *x* to *x* + *n* at time *t*. The person-year values can be found from the beginning and ending population values in a number of ways (Schoen, 1988, Chap. 4). Here we use the linear relationship

(2) \begin{eqnarray*}P{P}_{j}(x,n,t)=(n/2)[{P}_{j}(x,n,t)+{P}_{j}(x+n,n,t+n)]\end{eqnarray*}

that assumes that the *P*(*x*, *n*, *t*) function is linear between ages *x* and *x* + *n*. Even with 5-year age intervals, that linear assumption has been found to be satisfactory for most demographic work.

From Eqs. (1), straightforward algebra yields the solutions

\begin{eqnarray*}{m}_{\delta }(x,n,t)= \frac{{P}_{1}(x,n,t)+{P}_{2}(x,n,t)+{P}_{3}(x,n,t)-{P}_{1}(x+n,n,t+n)-{P}_{2}(x+n,n,t+n)-{P}_{3}(x+n,n,t+n)}{P{P}_{1}(x,n,t)+P{P}_{2}(x,n,t)+P{P}_{3}(x,n,t)} \end{eqnarray*}

\begin{eqnarray*}{m}_{12}(x,n,t)= \frac{[P{P}_{1}+P{P}_{3}][{P}_{2}(n)-{P}_{2}(0)]-P{P}_{2}[{P}_{1}(n)+{P}_{3}(n)-{P}_{1}(0)-{P}_{3}(0)]}{P{P}_{1}(x,n,t)[P{P}_{1}(x,n,t)+P{P}_{2}(x,n,t)+P{P}_{3}(x,n,t)]} \end{eqnarray*}

(3) \begin{eqnarray*}{m}_{13}(x,n,t)= \frac{[P{P}_{1}+P{P}_{2}][{P}_{3}(n)-{P}_{3}(0)]-P{P}_{3}[{P}_{1}(n)+{P}_{2}(n)-{P}_{1}(0)-{P}_{2}(0)]}{P{P}_{1}(x,n,t)[P{P}_{1}(x,n,t)+P{P}_{2}(x,n,t)+P{P}_{3}(x,n,t)]} \end{eqnarray*}

where the age and time identifiers have been dropped from the *PP*_*j*_ functions in the numerators and the age and time identifiers of the beginning and ending populations are simply denoted by (0) and (*n*), respectively, to simplify the presentation. The overall death rate is just the beginning number of persons minus the ending number, divided by the total number of person-years lived in the interval. The expressions for the transfer rates are a bit more complicated, but represent a weighted difference in state sizes over the interval, divided by the origin state number of person-years. Note that there is no assumption restricting a person to only one transfer during an interval (cf. Schoen, 1988, Ch. 4).

When the transfer rates take persons only from state 1 to state 2, state 2 to state 3, etc., the model can be termed “strictly” hierarchical. The flow equations of an *N* state strictly hierarchical model are

\begin{eqnarray*}{P}_{1}(x+n,n,t+n) & = & {P}_{1}(x,n,t)-P{P}_{1}(x,n,t)[{m}_{\delta }(x,n,t)+{m}_{12}(x,n,t)] \end{eqnarray*}

\begin{eqnarray*}{P}_{j}(x+n,n,t+n)\TFamp =\TFamp {P}_{j}(x,n,t)-P{P}_{j}(x,n,t)[{m}_{\delta }(x,n,t)+{m}_{j,j+1}(x,n,t)]\nonumber\\\displaystyle \TFamp \TFamp +\hspace*{1em}P{P}_{j-1}(x,n,t){m}_{j-1,j}(x,n,t) \end{eqnarray*}

(4) \begin{eqnarray*}{P}_{N}(x+n,n,t+n)\TFamp =\TFamp {P}_{N}(x,n,t)-P{P}_{N}(x,n,t){m}_{\delta }(x,n,t)\nonumber\\\displaystyle \TFamp \TFamp +\hspace*{1em}P{P}_{N-1}(x,n,t){m}_{N-1,N}(x,n,t)\end{eqnarray*}

where 1 < *j* < *N*. Those flow equations yield the death and transfer rates

(5a) \begin{eqnarray*}{m}_{\delta }(x,n,t)= \frac{{\mrm{\Sigma }}_{i}[{P}_{i}(x,n,t)-{P}_{i}(x+n,n,t+n)]}{{\mrm{\Sigma }}_{i}[P{P}_{i}(x,n,t)]} \end{eqnarray*}

(5b) \begin{eqnarray*}{m}_{j,j+1}(x,n,t)= \frac{{\mrm{\Sigma }}_{i}[{P}_{i}(x,n,t)-{P}_{i}(x+n,n,t+n)-P{P}_{i}(x,n,t){m}_{\delta }(x,n,t)]}{P{P}_{j}(x,n,t)} \end{eqnarray*}

where the sum in Eq. (5a) goes from 1 to *N*, and the sum in Eq. (5b) goes from 1 to *j*. The overall death rate, *m*_*δ*_(*x*, *n*, *t*), is again the difference between the beginning and ending populations divided by the total number of person-years lived in the interval. The transfer rate from state *j* to state *j* + 1 is the difference between the beginning and ending populations through state *j*, less the losses to death through state *j*, divided by the number of person-years lived in state *j* during the interval.

In general, the flow equations that describe the specific polytree form (or the structure of the desired multistate model), plus the initial and final population numbers in each state, can yield all of the transfer rates of a hierarchical multistate model. With the rates known, all model functions can readily be found (cf. Schoen, 1988). The only assumptions involved are that the population data are accurate, the population is closed, there is the same rate of death (or attrition) from every state, and the linear calculation assumption is appropriate. All of those assumptions can be relaxed with additional information. For example, if mortality is known, the interstate transfer rates can be found from cross-sectional surveys of the beginning and ending populations.

## Finding probabilities of transfer

The basic problem in life table construction is how to go from rates of transfer to probabilities of transfer. In multistate models, including hierarchical models, the problem is best approached by arraying the transfer rates in a matrix. With *N* living states, the *N* × *N* rate matrix can be written

(6) \begin{eqnarray*}\mathbf{M}(\mathbf{x},\mathbf{n},\mathbf{t})= \left[ \begin{array}{@{}lllll@{}} \displaystyle {\mrm{\Sigma }}_{j}{m}_{1j}&\displaystyle -{m}_{12}&\displaystyle -{m}_{13}&\displaystyle \cdots &\displaystyle -{m}_{1N}\\ \displaystyle -{m}_{21}&\displaystyle {\mrm{\Sigma }}_{j}{m}_{2j}&\displaystyle -{m}_{23}&\displaystyle \cdots &\displaystyle -{m}_{2N}\\ \displaystyle \cdots &\displaystyle \cdots &\displaystyle \cdots &\displaystyle &\displaystyle \cdots \\ \displaystyle -{m}_{N1}&\displaystyle -{m}_{N2}&\displaystyle -{m}_{N3}&\displaystyle \cdots &\displaystyle {\mrm{\Sigma }}_{j}{m}_{Nj} \end{array} \right] \end{eqnarray*}

where the interval identifiers have been dropped from the *m*’s and the sums over *j* in the diagonal elements exclude *m*_*jj*_ and include the rate to the dead state. In hierarchical models, only *N* transfer rates, including *m*_*δ*_, are nonzero.

There are a number of ways to calculate the array of transition probabilities from **M**. One is to assume that the underlying risks of transition are constant over the interval and find the probabilities by exponentiating **M** (cf. Schoen, 2006b, Chap.1). Here, we will continue to use the linear assumption underlying Eq. (2), and employ the general linear solution of Rogers & Ledent (1976, Eq. (2)), as written in Schoen (2006b, Chap. 1). We then have

(7) \begin{eqnarray*}\mbrm{\Pi }(\mathbf{x},\mathbf{n},\mathbf{t})=[\mathbf{I}-(n/2)\mathbf{M}(\mathbf{x},\mathbf{n},\mathbf{t})][\mathbf{I}+(n/2)\mathbf{M}(\mathbf{x},\mathbf{n},\mathbf{t})]^{-1}\end{eqnarray*}

where **I** is the *N* × *N* identity matrix. Transition probability matrix **Π**(**x**, **n**, **t**) is an *N* × *N* matrix whose *ij*th element is *π*_*ij*_(*x*, *n*, *t*), the probability that a person in state *i* at age *x* and time *t* will be in state *j* exactly *n* years later. In hierarchical models, **Π** can be written as an upper triangular matrix, that is a matrix where all of the elements below the main diagonal are zero. That can be accomplished by numbering the states so that every nonzero *m*_*ij*_ has *i* < *j*.

The age-specific transition probability matrices for a period allow the construction of a multistate life table for that period. That life table provides, among other functions, the number of persons in each state at every age. Those life table survivorship values follow from the projection relationship

(8) \begin{eqnarray*}{\boldsymbol{\ell }}^{\mathbf{T}}(\mathbf{x}+\mathbf{n})={\boldsymbol{\ell }}^{\mathbf{T}}(\mathbf{x})\mbrm{\Pi }(\mathbf{x},\mathbf{n},\mathbf{t})\end{eqnarray*}

where the *j*th element of the *N* element survivorship vector **ℓ**(**x**) is ℓ_*j*_(*x*), the number of persons in the life table cohort who are in state *j* at exact age *x*. The superscript **T** indicates that column vector **ℓ**(**x**) is transposed into a row vector. The initial (radix) vector, **ℓ**(**0**), is assumed to be known. The remaining multistate life table functions follow in the usual manner (cf. Schoen, 1988, Chap. 4).

The calculation that provides the transition probabilities from observed adjacent population values proceeds from the observed populations to the rates of transfer and then to the transition probabilities. It is thus rate centered, not probability based. Rates are preferable because they directly reflect the underlying behavior, i.e., the risk of movement from state *i* to state *j*, while probabilities only reflect that behavior in the context of other prevailing risks. In the hierarchical model, there are only *N* − 1 nonzero rates of transfer between living states while, because of multiple moves within an interval, there can be *N*(*N* − 1)∕2 nonzero probabilities of transfer. That larger (for *N* > 2) number of probabilities cannot be determined from adjacent population data alone, unless the underlying rate structure is hierarchical. The proposed rate based approach accommodates multiple moves with an interval via the underlying Markov assumption that the risk of transfer depends only on current age and state.

## An application to parity status life tables

The procedure described in the previous two sections can find the rates and probabilities of any hierarchical multistate model from initial and final population data alone. To illustrate the procedure numerically, let us consider an important demographic application: the parity status model.

### The demographic significance of parity

Parity, the number of live births a woman has had, is a meaningful demographic indicator. Its importance as a fertility measure was recognized in the 1950s (e.g., Henry, 1953; Whelpton, 1954), and by the 1960s it was incorporated into population projections (cf. Akers, 1965). Parity is associated with subsequent fertility as well as with many other social, economic, and demographic behaviors. Childlessness has been a particular focus of interest, and has been seen as having strong connections to core social values (Bulcroft & Teachman, 2004; Koropeckyj-Cox & Pendell, 2007; Veevers, 1980).

Data on births by age and parity are now frequently published by national statistical agencies (e.g., Heuser, 1976). Life tables recognizing parity status go back at least to Chiang & Van Den Berg (1982). Previous research has shown that parity changes were important in understanding fertility fluctuations (e.g., Ryder, 1969), and that the ultimate parity distributions of American women have varied greatly. A parity status life table based on 1970 fertility rates implied that 12% of women would remain childless, while 25% would have 4 or more children. Under 1995 rates, the life table proportion childless increased to 15%, while the proportion with 4 or more children dropped to 14% (Schoen, 2006a, Table 1). Recent population data on levels of childlessness among American women aged 40–44 indicate that 18% were childless in 1995 and 15% in 2002 (Abma & Martinez, 2006).

**Table 1:** Percentages of women by age and parity, United States 2005 and 2010.

Age	Parity							
	0	1	2	3	4	5	6	Total
**YEAR 2005**								
10–14	100	0	0	0	0	0	0	100
15–19	93.35	4.55	1.45	0.55	0.10	0	0	100
20–24	68.65	18.55	9.25	2.65	0.70	0.10	0.1	100
25–29	44.90	23.15	20.05	8.30	2.65	0.55	0.4	100
30–34	26.90	21.35	29.05	15.55	4.90	1.35	0.9	100
35–39	19.25	18.20	34.85	18.40	6.15	1.75	1.4	100
**YEAR 2010**								
15–19	94.7	4.4	0.6	0.3	0	0	0	100
20–24	70.5	18.1	9.0	2.0	0.3	0.1	0	100
25–29	47.6	22.7	18.7	7.8	2.3	0.6	0.3	100
30–34	29.7	19.2	29.1	14.3	5.2	1.5	1.0	100
35–39	19.7	18.5	32.7	19.7	5.9	2.1	1.4	100
40–44	18.8	18.5	33.3	19.1	6.8	1.9	1.6	100

**Notes.**

Note: percentages for 2005 are the average of those in 2004 and 2006.

Source: Census.gov/hhes/fertility/data/cps/2004.html, Census.gov/hhes/fertility/data/cps/2006.html and Census.gov/hhes/fertility/data/cps/2010.html, downloaded 9/11/2014.

### US parity distributions and the rates they imply

The age distribution of American women, by number of children ever born, is available for even numbered years from the Census Bureau’s Current Population Survey, accessible online at http://www.census.gov/hhes/fertility/data/cps/. Table 1 shows the proportions of women by age and parity for 2005 and 2010, the former year found as the average of the values for 2004 and 2006. Entries have been rounded so that all rows sum to exactly 100. There were very few women at parities higher than 6, so 6 was taken to be the highest parity possible. In 2010, 18.8% of women aged 40–44 were childless, while 1.6% attained parity 6.

The figures in Table 1, along with the assumption of no mortality at the reproductive ages, is sufficient to allow the construction of a parity status life table for the 2005–2010 interval. The model is strictly hierarchical, as women of parity *j* can only move to parity *j* + 1. The transfer rates of the model follow from Eq. (5), and are shown in Table 2. The age intervals shown are offset by 2.5 years from those in Table 1, as *P*_*j*_(*x*, *n*, *t*) was taken to be the number of persons at exact age *x* + *n*∕2. Fertility rates are assumed to be zero for women past age group 40–44 in 2010. That assumption, while dictated by the available data, is not problematic because the rates at ages 37.5–42.5 are already quite small, and the rates at higher ages are even smaller.

**Table 2:** Rates of movement to successive parities, by age, US women 2005–10.

Age	Rate of movement from parity *j* to parity *j* + 1 (*m*_*j*,*j*+1_)					
	*m*_01_	*m*_12_	*m*_23_	*m*_34_	*m*_45_	*m*_56_
12.5–17.4	.01089	.08182	.20000	0	0	0
17.5–22.4	.05620	.16777	.07464	.07843	.30000	.30000^a^
22.5–27.4	.07243	.16388	.10662	.08804	.09333	.11429
27.5–32.4	.08150	.18087	.08220	.07257	.07898	.11707
32.5–37.4	.06180	.10088	.04146	.02553	.04630	.05797
37.5–42.4	.00473	.00163	.00998	.01067	.01081	.02192

**Notes.**

a Adjusted value; calculated rate of 0.8 based on very few exposures.

Source: calculated from figures in Table 1 as described in text.

The rates of transfer to parities 1 and 2 reach their maximum at ages 27.5–32.5, while the rates to parities 3 and 4 reach their maximum at ages 22.5–27.5. At young ages, rates to the highest parities are based on few observations, and tend to be unstable (e.g., the rate to parity 6 at ages 17.5–22.5). However, those rates are applied to the very small numbers of persons at those high parities, and hence have little impact on the life table parity distributions.

### Calculating the transition probabilities and life table survivorship

Using Eq. (7), the rates of transfer can be used to calculate transition probability matrices for every age. Because the model is strictly hierarchical, the elements of the **Π** matrices are patterned, and can be written out in scalar form. The *j*th diagonal element has the basic life table survivorship form

(9) \begin{eqnarray*}{\pi }_{jj}=[2-n\hspace*{1em}{m}_{j-1,j}]/[2+n\hspace*{1em}{m}_{j-1,j}].\end{eqnarray*}

The element in the *j*th column (1 < *j* < *N*) of the first row is given by

(10) \begin{eqnarray*}{\pi }_{{1}_{j}}=[4{n}^{j-1}{\mbrm{ \Pi }}_{i-1}^{j-1}{m}_{i-1,i}]/[{\mbrm{\Pi }}_{i-1}^{j}(2+n\hspace*{1em}{m}_{i-1,i})].\end{eqnarray*}

In general, *π*_*i*+*k*,*j*+*k*_, the element in the (*i* + *k*)th row and (*j* + *k*)th column (*i* + *k* < *j* + *k* < *N*), equals *π*_1*j*_ with all rate indexes increased by *k*. For example, in the parity status life table with *n* = 5, the probability of going from parity 2 to parity 4 is given by

\begin{eqnarray*}{\pi }_{24}={\pi }_{13}[\text{all rate indexes increased by 1}] \end{eqnarray*}

or

(11) \begin{eqnarray*}{\pi }_{24}=[100\hspace*{1em}{m}_{12}{m}_{23}]/\{[2+5\hspace*{1em}{m}_{12}][2+5\hspace*{1em}{m}_{23}][2+5\hspace*{1em}{m}_{34}]\}.\end{eqnarray*}

The elements of **Π** in the *N*th column are different because that state has no movements (decrements) to another state. Equation (7) yields

(12) \begin{eqnarray*}{\pi }_{1N}=[2{n}^{N-1}{\mbrm{ \Pi }}_{i=1}^{N-1}{m}_{i-1,i}]/[{\mbrm{\Pi }}_{i=1}^{N-1}(2+n\hspace*{1em}{m}_{i-1,i})]\end{eqnarray*}

which differs from Eq. (10) in that the initial factor is 2 rather than 4, and that the product in the denominator goes to *N* − 1 instead of *N*. The element in the *i*th row (*i* > 1) and *N*th column is similarly the expression given in Eq. (11), but with an initial factor of 2 and a denominator product ending with the *N* − 1st term.

Under the linear assumption, the expressions for the probabilities of moving more than one state in strictly hierarchical models can be interpreted in terms of successive risks of transfer. The probability of moving from state *j* − 1 to state *j* is [*nm*_*j*−1,*j*_]∕[1 + (*n*∕2)*m*_*j*−1,*j*_]. The linear assumption implies that moves occur uniformly over the interval or, on average, at the midpoint, *n*∕2. The movers are then exposed to the risk of moving from state *j* to state *j* + 1 according to rate *m*_*j*,*j*+1_ over half the interval. The successive factors in Eq. (10) reflect the likelihood of those additional moves, each with a shorter time exposure to risk.

### The parity status life table

At birth, all women are at parity zero, so the parity distribution at initial age 12.5 has one person at parity zero and zero persons at al higher parities. The life table parity distributions at successively higher ages are found using projection Eq. (8).

Table 3 shows the parity distributions at ages 12.5 through 42.5 for US Women based on the data for 2005–2010. The proportion at parity zero steadily decreases, while the proportions at parities 3 and above steadily increase. The proportion at parity 1 reaches a peak at age 27.5, while the proportion at parity 2 peaks at age 37.5. At age 42.5, 23.4% of women are still at parity zero, while only 8.6% have 4 or more children. The US proportion childless has continued to rise, and is approaching one out of every four women. In contrast, large families of 4 or more children characterize barely one woman in twelve.

**Table 3:** Parity distributions and cumulated fertility, by age, parity status life table for US women 2005–10.

Age	Proportion of women at parity indicated							Cumulated fertility to age indicated
	0	1	2	3	4	5	6
12.5	1.	0	0	0	0	0	0	0
17.5	.947	.044	.006	.003	0	0	0	.06
22.5	.714	.182	.084	.016	.002	.001	.001	.42
27.5	.495	.232	.183	.069	.016	.004	.002	.90
32.5	.327	.203	.283	.129	.041	.011	.006	1.41
37.5	.240	.191	.320	.172	.050	.017	.010	1.69
42.5	.234	.195	.306	.178	.056	.018	.012	1.73

**Notes.**

Cumulated fertility is the sum of the births to the life table cohort (of 1 woman) up to the given age. Parity 6 is assumed to be the highest parity possible, and no fertility is assumed after age 42.5. Thus cumulated fertility to age 42.5 represents the Parity Status Total Fertility Rate.

Source: calculated from Table 2 as described in text.

Many parity-specific analyses examine parity progression ratios (PPRs). The PPR from parity *j* to parity *j* + 1 is the proportion of persons who attain parity *j* who go on to attain parity *j* + 1. PPRs can be calculated from the ℓ_*j*_ functions. For example, at age 42.5, the PPR from parity 2 to parity 3 is the sum of the ℓ_*j*_ values for parity 3 and above divided by the sum of the ℓ_*j*_ values for parity 2 and above, or .264/.570 = .463. That PPR is considerably higher than the PPR from parity 3 to parity 4, which is .326.

The Parity Specific Total Fertility Rate (PSTFR) of the life table cohort can be found from the parity distribution of women at the end of childbearing, here taken to be age 42.5. Algebraically,

(13) \begin{eqnarray*}\mathrm{PSTFR}={\mathop{\Sigma }\nolimits }_{j=1}^{N}(j-1){\ell }_{j}(42.5)\end{eqnarray*}

where Σℓ_*j*_(42.5) = 1 and state 1 is parity 0. The PSTFR differs from the conventional TFR because it is calculated from age-parity-specific fertility rates, rather than the usual age-specific fertility rates, and thus takes the parity composition of the hypothetical cohort into account. Table 3 shows a PSTFR, or cumulative fertility to age 42.5, of 1.73, less than two children per woman.

The life table proportion childless, 0.234, is substantially larger than 0.188, the proportion childless among American women in 2010. That observed proportion reflects childlessness in one actual cohort of women. In contrast, the life table proportion indicates that a greater fraction of women would never have a child under the age-parity-specific fertility rates prevailing during 2005–2010. The initiation of childbearing in the observed cohort followed a different pattern from that in the 2005–2010 period.

## Summary and conclusions

Many demographic phenomena can be represented by hierarchical models, that is by models with *N* living states that have *N* − 1 rates of movement between them. In such models, the rates of transfer between living states and to death can be found from data on the number of persons in each state at the beginning and end of a given interval. With the rates of transfer provided by Eq. (5), the probabilities of transfer can be found from Eq. (7) and used to produce a multistate life table. Exploiting the ability of population counts to infer rates of transfer in hierarchical contexts broadens the scope of demographic analysis.

The potential of the approach is illustrated by an application to parity status. A parity status life table for US Women, 2005–2010, is constructed from survey data on the fraction of women, by age, in each parity status in 2004, 2006, and 2010. The results show that the US proportion childless is continuing to increase, with nearly a quarter of women aged 42.5 remaining at parity zero. The 2–4 child pattern of the past has been replaced by a 0–3 child pattern.

##  Supplemental Information

10.7717/peerj.2535/supp-1 Supplemental Information 1 Number of women by number of children ever born from the June 2004 Current Population Survey conducted by the US Census Bureau

10.7717/peerj.2535/supp-2 Supplemental Information 2 Number of women by number of children ever born from the June 2006 Current Population Survey conducted by the US Census Bureau

10.7717/peerj.2535/supp-3 Supplemental Information 3 Number of women by number of children ever born from the June 2010 Current Population Survey conducted by the US Census Bureau

## Additional Information and Declarations

### Data Availability

The following information was supplied regarding data availability: The raw data has been supplied as Supplementary Files.
